# Intermittent parathyroid hormone improve bone microarchitecture of the mandible and femoral head in ovariectomized rats

**DOI:** 10.1186/s12891-017-1530-4

**Published:** 2017-04-24

**Authors:** Ying-Ju Chen, Shun-Ping Wang, Fu-Chou Cheng, Pei-Yu Hsu, Yu-Fen Li, Jay Wu, Heng-Li Huang, Ming-Tzu Tsai, Jui-Ting Hsu

**Affiliations:** 1 Department of Food and Nutrition, Providence University, Taichung, 433 Taiwan; 2 Department of Orthopaedics, Taichung Veterans General Hospital, Taichung, 407 Taiwan; 3 Stem Cell Medical Research Center, Department of Medical Research, Taichung Veterans General Hospital, Taichung, 407 Taiwan; 4 Department of Biomedical Imaging and Radiological Science, China Medical University, Taichung, 404 Taiwan; 5 Institute of Biostatistics, China Medical University, Taichung, 404 Taiwan; 6 Department of Biomedical Imaging and Radiological Sciences, National Yang-Ming University, Taipei, 112 Taiwan; 7 School of Dentistry, College of Medicine, China Medical University, 91 Hsueh-Shih Road, Taichung, 40402 Taiwan; 8 Department of Bioinformatics and Medical Engineering, Asia University, Taichung, 413 Taiwan; 9 Department of Biomedical Engineering, Hungkuang University, Taichung, 433 Taiwan

**Keywords:** Parathyroid hormone, Trabecular bone, Microcomputed tomography (micro-CT), Mandible, Femoral head

## Abstract

**Background:**

Intermittent parathyroid hormone (PTH) can be used to treat osteoporosis of the spine and hip. However, whether it can be used to treat osteoporosis of the mandible is unclear. The purpose of this study was to explore the influence of applying intermittent PTH to ovariectomized rats on the trabecular bone microarchitecture of the mandible and femoral head.

**Methods:**

Eighteen female rats were divided into three groups: the healthy group, ovariectomized (OVX) group, and OVX + PTH group. The OVX group and OVX + PTH group had an OVX at 8 weeks of age. The OVX + PTH group received intermittent PTH therapy for 12 weeks. The mandibles and femurs of all rats were removed at 20 weeks and were then scanned using microcomputed tomography (micro-CT).

**Results:**

From the micro-CT analysis, the trabecular bone microarchitecture of the mandible and femoral head are offered as follows: (1) The bone volume fraction and trabecular thickness in the OVX group were lower than those in the healthy group. (2) The bone volume fraction and trabecular thickness in the OVX + PTH group approximated those in the healthy group.

**Conclusion:**

The conclusions of this study regarding the trabecular bone microarchitecture of the mandible and femoral head are offered as follows: (1) The BV/TV and TbTh in the OVX group were lower than those in the healthy group. (2) The BV/TV and TbTh in the OVX + PTH group approximated those in the healthy group, therefore, intermittent PTH displayed high efficacy for treating femoral or mandibular deterioration of bone microstructure resulting from loss of ovarian function. Osteoporosis of the femur or mandible in the rats was ameliorated by intermittent PTH therapy.

## Background

As the average human life expectancy increases, osteoporosis has become a major health concern. According to the World Health Organization (WHO), women are considered to have osteoporosis if their bone mineral density (BMD) is less than or equal to 2.5 standard deviations of the mean BMD of female adults according to dual-energy X-ray absorptiometry (DXA) measurements [1]. Clinically, osteoporosis is categorized into two types: primary osteoporosis and secondary osteoporosis. Furthermore, there are two types of primary osteoporosis (types I and II). Type I primary osteoporosis refers to postmenopausal osteoporosis, which occurs because women’s ovaries stop functioning following menopause and their estrogen production reduces. Type II primary osteoporosis is senile osteoporosis, which occurs in elderly people when the body can no longer synthesize sufficient active Vitamin D_3_, leading to reduced calcium absorption, which results in bone resorption and increases the risk of bone fracture. Secondary osteoporosis often occurs at various age levels in men and women and is mainly caused by endocrine disease, blood disease, malnutrition, drugs, or other diseases. Regarding the prevalence of osteoporosis, the Census and National Health and Nutrition Examination Survey (2005–2010) indicated that in 2010, 10.2 million American people aged ≥50 years had osteoporosis; moreover, 43.4 million people reportedly had low BMD and were at risk of osteoporosis [2]. Previous studies have indicated that 30% of menopausal women in the United States and Europe had osteoporosis. In particular, 40% of these women were predicted to experience multiple fragility fractures during their remaining lifetime [3]. Fragility fractures mainly occur in parts of body that contain more trabecular bones (e.g., the spine, wrist, and hip) [4].

Several studies have investigated the correlation between ovariectomized (OVX) and osteoporosis in menopsausal women or in animals. Previous studies involving animal experiments have indicated that systemic osteoporosis was highly correlated with bone loss of the jawbone. Osteoporosis not only increases the incidence of bone fractures in the hip, spine, and wrist of patients, but also causes tooth loss and degradation of the alveolar process, which supports the teeth [5]. Dental implants have become an effective method for treating tooth loss; successful dental implantation is highly correlated with the quality of the alveolar bone [6]. Therefore, exploring osteoporosis of the mandible and improving the pharmaceutical treatment of osteoporosis are crucial.

Currently, two drug types are commonly used to treat osteoporosis. One type is antiresorptive drugs, which inhibit osteoclasts [7]. In particular, bisphosphonate drugs are the most commonly used type of antiresorptive drug. Previous studies have indicated that bisphosphonate drugs can effectively slow osteoporosis of the hip and spine. However, some studies have reported that using bisphosphonate drugs for a long period can induce adverse effects such as gastrointestinal intolerance, esophageal cancer, atypical fractures, atrial fibrillation, acute reaction, ocular inflammation, or osteonecrosis of the jaw [8]. The other type of drugs is anabolic drugs, which can activate osteoblasts. Intermittent administration of parathyroid hormone (PTH) is the most common method used for delivering anabolic drugs. Anabolic bone therapy can increase the number and activity of osteoblasts and reduce the likelihood of apoptosis [9, 10]. In addition, intermittent PTH can effectively treat osteoporosis of the hip and spine [9–11]. However, only a few studies have explored the influence of intermittent PTH on the mandible [12–14]. Therefore, whether intermittent PTH can ameliorate destruction of the jawbone related to estrogen deficiency is worthy of investigation. Previous studies have often used ovariectomized rats to investigate osteoporosis resulting from decreased estrogen secretion in menopausal women because the U.S. Food and Drug Administration approved using ovariectomized rats as a clinical model [15].

Intermittent PTH can be used to treat osteoporosis of the spine and hip. However, whether it can be used to treat osteoporosis of the mandible is unclear. The purpose of this study was to explore the influence of applying intermittent PTH to ovariectomized rats on the trabecular bone microarchitecture of the mandible and femoral head.

## Methods

### Animal preparation and experimental design

In this study, 18 Wistar female rats were used for an animal experiment. The rats were divided into three groups (healthy, ovariectomy [OVX], and OVX + PTH) with six rats in each group. (1) The healthy group did not receive OVX and PTH intervention. (2) The OVX group received an OVX at 8 weeks of age. (3) The OVX + PTH group received an OVX when they were 8 weeks old; subsequently, they were injected with 50 μg/kg of teriparatide (recombinant human PTH [1–34], Eli Lilly and Co., IN, USA) three times per week for 12 weeks. All rats were purchased from BioLASCO Taiwan Co., Ltd. (Taipei, Taiwan). Rats were housed in a temperature (25 °C) and light-controlled room (12∶12 h light–dark cycle), standard rat chow, and water ad libitum. In addition, all rats were weighed weekly since 8 weeks of age. At 20 weeks, these experimental animals were sacrificed by carbon dioxide asphyxiation, the entirety of the right mandible and femur were harvested from every rat within 20 min. All bones removed were covered with a gauze moistened with a 0.9% saline solution and were then preserved at−20 °C. All the animal experimental procedures were approved by the Research Ethics Committee of the Taichung Veterans General Hospital (Permit Number: La1031191) and were performed in accordance with their Guidelines for the Care and Use of Laboratory Animals.

### Micro-computed tomography measurement

**Fig. 1:**
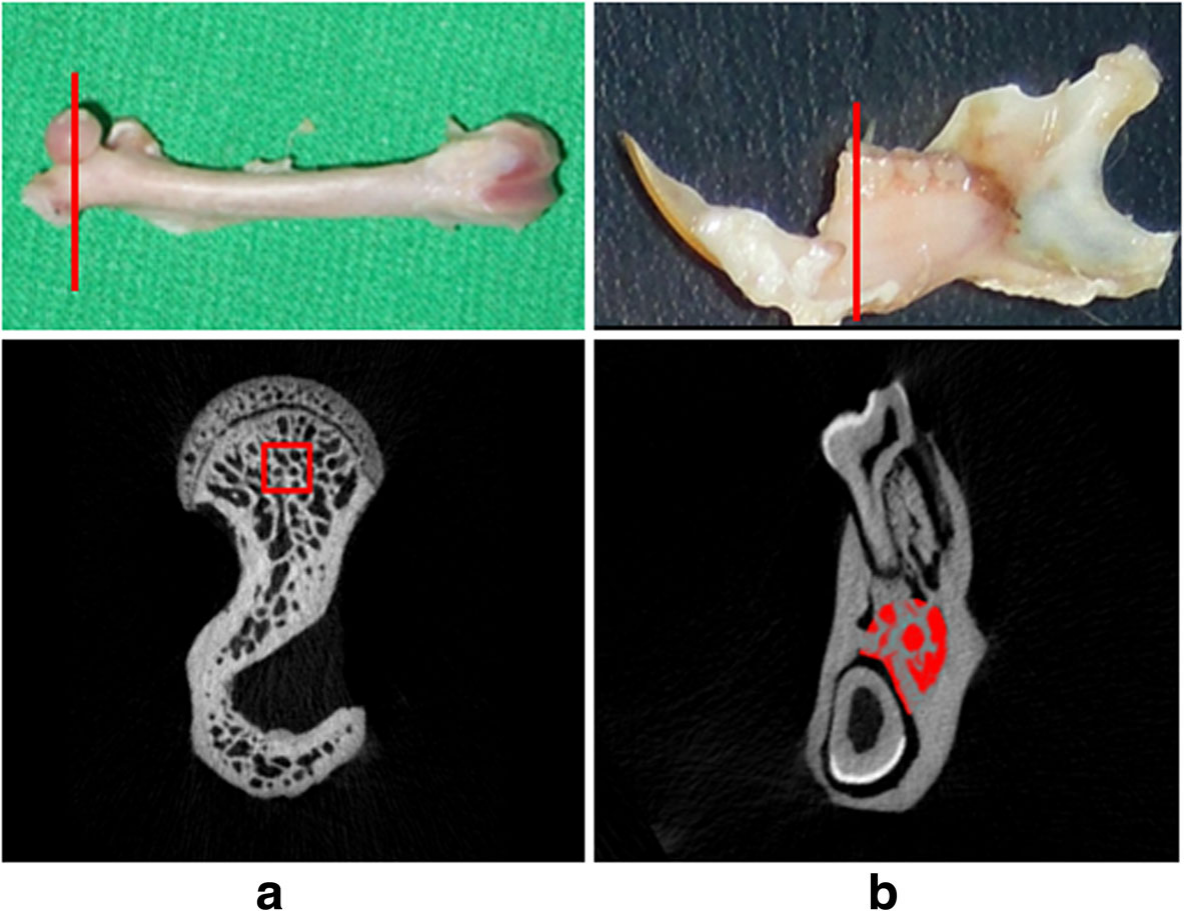
Bone photographs and micro-CT images: **a** intact right femur (*left*), cross-sectional slices displaying the structure of the middle femoral head (*right*), **b** whole mandible (*left*), cross-sectional slices displaying the structure of the middle first molar (*right*)

Microcomputed tomography (micro-CT) images of each femur and mandible were obtained using a micro-CT device (Skyscan 1076, Skyscan, Aartselaar, Belgium). The scanning parameters were set at 49 kV, 200 μA, 500 ms, and the voxel resolution was set at 18.27 μm. The micro-CT images were imported into CTAn software (Skyscan) to measure the following four parameters of the trabecular bone microarchitecture: bone volume fraction (bone volume/total volume [BV/TV]; unit = %), trabecular bone thickness (TbTh; unit = mm), trabecular bone separation (TbSp; unit = mm), and trabecular bone number (TbN; unit = 1/mm) of the femoral head (40 × 40 × 40 voxels; approximating 0.73 × 0.73 × 0.73 mm^3^), and the region below the mandibular first molar (Fig. 1). The trabecular bone was segmented with a fixed threshold value of 65, which is the automated average threshold determined by CTAn Software (Skyscan) for all rats.

### Statistical analysis

The four trabecular bone microarchitecture parameters of the mandible and femoral head in the three groups were summarized as median (interquartile range [IQR]). The Kruskal–Wallis test was used to compare the difference in body weight, BV/TV, TbTh, TbSp, and TbN among the three groups in the mandible and femoral head. Post hoc pairwise comparisons were conducted using the Mann–Whitney exact tests with the Bonferroni adjustment, and the significance level was set at 0.0167 (0.05/3). No power analysis for the sample size was performed in advance. However, the power analysis was carried out for post hoc pairwise comparisons for the present sample size. All statistical analyses were performed using SPSS Version 19 (IBM Corporation, Armonk, NY, USA).

## Results

### Body weight

**Fig. 2:**
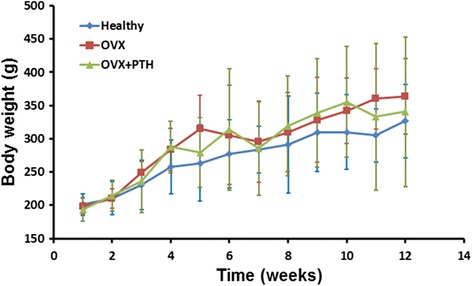
Body weight (median ± interquartile range) of the three groups throughout the experimental period. Each group include six rats. (♦: *blue* = healthy; ■: *red* = OVX; ▲: *green* = OVX + PTH)

Figure 2 shows the median weights of the three groups throughout the experiment. Prior to the experiment (i.e., when the rats were 8 weeks old), no significant difference was observed in weight among the three groups. The trend in the median weight of the rats throughout the 12-week experiment indicated that the healthy group was the lightest, whereas the OVX group was the heaviest among the three groups. Except at Week 11, the OVX group was significantly heavier than the healthy group (*p* = .002); for all other time points, no significant difference in weight was observed among the three groups.

### Trabecular bone microarchitecture

**Fig. 3:**
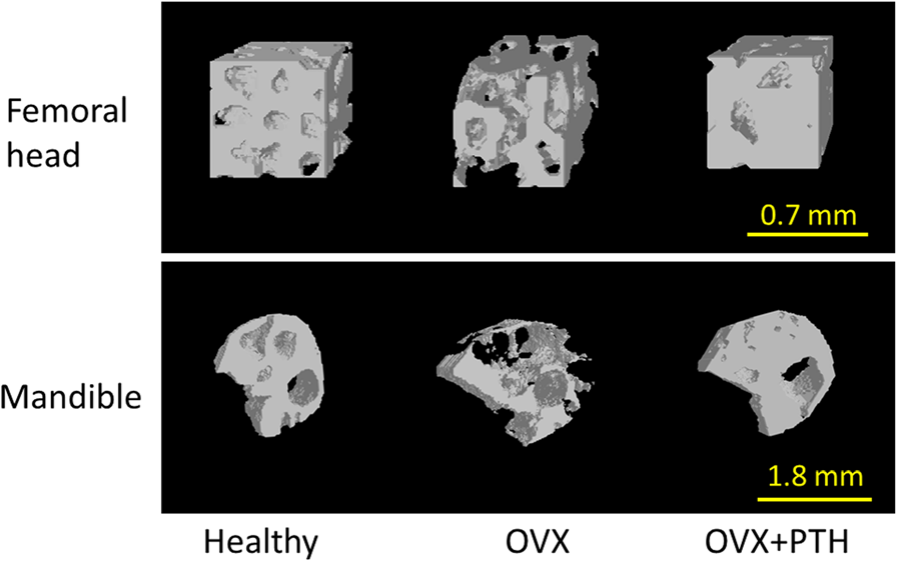
Three-dimensional images of the trabecular bone microarchitecture in the femoral head (region in the center of femoral head) and mandible (region below the mandibular first molar) from the three groups: healthy group, ovariectomized (OVX) group, and OVX+ parathyroid hormone (PTH) group. Each group includes six rats

Table 1 shows the measurement results related to the trabecular bone microarchitecture of the mandible and femoral head. In both structures, the 3D diagram shows that the OVX and OVX + PTH groups respectively had the lowest and highest BMD in the trabecular bone microarchitecture (Fig. 3). The results accord with the BV/TV tendency in the three groups. In both the mandible and femoral head, the BV/TV of the OVX group was the lowest among all three groups. In addition, the median for the OVX + PTH group was higher than that for the healthy group. However, no significant difference was observed in the BV/TV of the mandible or femoral head between the healthy and OVX + PTH groups.

**Table 1:** Measurement results of the trabecular bone microarchitecture of the mandible and femoral head in the three groups: healthy group, ovariectomized (OVX) group, and OVX+ parathyroid hormone (PTH) group

Parameters (unit)	Value	Mandible				Femoral head			
		Healthy	OVX	OVX + PTH	*P*†	Healthy	OVX	OVX + PTH	*P*†
BV/TV (%)	Median*	51.704 ^a^	38.486 ^b^	52.398 ^a^	0.003	60.278 ^a^	42.276 ^b^	63.317 ^a^	0.013
	IQR	6.253	9.111	6.399		11.329	12.496	13.125	
	Max	59.420	43.084	60.530		75.871	47.726	64.612	
	Min	49.154	30.052	49.310		54.668	27.911	46.626	
TbTh (mm)	Median*	0.239 ^a^	0.193 ^b^	0.203 ^ab^	0.047	0.118 ^a^	0.104 ^b^	0.129 ^a^	0.023
	IQR	0.028	0.070	0.042		0.017	0.029	0.017	
	Max	0.267	0.246	0.251		0.159	0.116	0.148	
	Min	0.220	0.146	0.156		0.114	0.081	0.106	
TbSp (mm)	Median*	0.285 ^ab^	0.322 ^a^	0.278 ^b^	0.008	0.117 ^a^	0.148 ^b^	0.117 ^ab^	0.046
	IQR	0.041	0.047	0.072		0.036	0.023	0.036	
	Max	0.333	0.436	0.303		0.139	0.178	0.147	
	Min	0.261	0.305	0.219		0.098	0.128	0.103	
TbN (1/mm)	Median*	2.206 ^a^	2.078 ^a^	2.471 ^a^	0.016	4.915 ^a^	4.068 ^b^	4.518 ^ab^	0.008
	IQR	0.108	0.429	0.793		0.487	0.456	0.712	
	Max	2.241	2.188	3.407		5.560	4.376	5.008	
	Min	2.055	1.679	2.058		4.586	3.463	4.114	

Each group includes six rats

*BV/TV* (bone volume/total volume) bone volume fraction, *TbTh* trabecular bone thickness, *TbSp* trabecular bone separation, *TbN* trabecular bone number, *IQR* interquartile range, *Max* maximum, *Min* minimum, *OVX* ovariectomy, *PTH* parathyroid hormone

†Kruskal–Wallis test

* Post hoc pairwise comparisons were conducted using Mann–Whitney exact tests with the Bonferroni adjustment; medians with the same letter (a or b) are not significantly different at the 0.0167 (0.05/3) level

The TbTh and BV/TV tendencies were similar for all three groups. Specifically, the TbTh in the healthy and OVX + PTH groups was greater than that in the OVX group. A significant difference was observed in the TbTh of the mandible between the healthy and OVX groups, but the differences between the OVX + PTH and healthy groups and between the OVX + PTH and OVX groups were nonsignificant.

A significant difference was observed in the TbSp of the mandible between the OVX and OVX + PTH groups; however, the differences between the healthy and OVX groups and between the healthy and OVX + PTH groups were nonsignificant. Regarding the femoral head, the difference in TbSp between the healthy and OVX groups was significant; however, no significant difference was observed between the OVX + PTH and healthy groups or between the OVX + PTH and OVX groups.

The TbN in the mandible was the lowest in the OVX group and highest in the OVX + PTH group; however, the TbN differed nonsignificantly among the three groups. For the femoral head, the TbN and TbSp tendencies were similar. A significant difference in TbN was observed between the healthy and OVX groups, but the differences between the OVX + PTH and healthy groups and between the OVX + PTH and OVX groups were nonsignificant.

## Discussion

As the number of elderly people increases, osteoporosis will become more prevalent. Patients with osteoporosis are at risk of bone fractures, which invariably reduces their quality of life. Regarding anabolic drugs, previous studies have indicated that intermittent PTH effectively enhances BMD; however, most studies have focused on the vertebra body or long bones, and few studies have investigated the influence of intermittent PTH on the trabecular bone microarchitecture of the mandible. This study is the first one to use micro-CT to explore OVX-induced bone change in rats to determine how intermittent PTH alters the trabecular bone microarchitecture in the femoral head and mandible. The present experimental results show that intermittent PTH was highly effective for treating bone osteoporosis of the femur and mandible caused by the loss of ovarian function.

Numerous clinical studies have explored the relationship between systemic skeletal bone density and jawbone density through using various methods; hence, the results of such studies have varied considerably. Some epidemiological studies have indicated that systemic osteoporosis is positively correlated with mineral density in the mandible [16, 17], whereas others have shown that systemic BMD is uncorrelated with the resorption of the edentulous jawbone [18, 19]. Consequently, no consensus has been reached regarding the correlation between systemic osteoporosis and bone loss in the jaws. However, in recent years, researchers have considered that the jawbone is likely influenced by systemic osteoporosis [20, 21].

Several previous studies involving rat experiments have indicated that female rats develop systemic skeletal osteoporosis and jawbone deterioration in associated with estrogen deficiency following an OVX. In some studies, female rats of various ages have undergone an OVX and then exhibited a change in their BMD over time. Sengupta et al. [22] indicated that rats are sexually mature at 6 weeks of age. Johnston and Ward [23] indicated that 12 weeks post-OVX was the optimal time for simulating postmenopausal bone loss. Therefore, in the present study, rats underwent an OVX at 8 weeks of age, and the change in the trabecular bone microarchitecture of their femurs and mandibles were observed at 12 weeks post-OVX.

Numerous methods have been used to assess bone mass (e.g., DXA [24], CT [25], quantitative computed tomography [QCT] [24], peripheral QCT [26], quantitative ultrasound [27], and dental cone-beam CT (dental CBCT) [28, 29]. Micro-CT should be the optimal method for clearly observing the trabecular bone microarchitecture, and has been considered as a gold standard for assessing bone morphology and microstructure. Moreover, it can be used to measure numerous parameters of the trabecular bone microarchitecture. Bouxsein et al. [30] indicated that BV/TV, TbTh, TbSp, and TbN are four representative parameters of the trabecular bone microarchitecture.

Previous studies have explored the differences in the trabecular bone microarchitecture of the femur between ovariectomized and normal rats. Washimi et al. [31] reported significant differences in the four parameters of the trabecular bone microarchitecture of the distal femoral metaphysis, as follows: BV/TV = −58.1%, TbTh = −13.0%, TbSp = +200.0%, and TbN = −56.9%. Similarly, our experimental results revealed significant differences in these parameters of the trabecular bone microarchitecture of the femoral head, as follows: BV/TV = −29.9%, TbTh = −11.9%, TbSp = +26.5%, and TbN = −17.2%. Leitner et al. [32] measured BV/TV for the distal femoral metaphysis in rats and found that the BV/TV in an OVX group decreased by 36.8% compared with that for the healthy group. The results obtained in the present study differ from previous studies may be related to the species or age of the rats, or the post-OVX duration before measurements were performed. Another reason may be related to the regions measured by micro-CT. Previous studies have measured the trabecular bone microarchitecture of the distal femoral metaphysis [31, 32], whereas the trabecular bone microarchitecture of the femoral head was measured in the present study. Despite these differences, similar to previous studies, we observed that the BMD in the trabecular bone of the OVX group was significantly lower than that in the healthy group.

In this study, among the parameters of the trabecular bone microarchitecture of the mandible, significant differences in BV/TV and TbTh were observed between the OVX and healthy groups (BV/TV = −25.6% and TbTh = −19.3%), but no significant difference in TbSp or TbN was observed between these two groups. In measuring the trabecular bone microarchitecture of the interradicular septum of the mandibular first molar, Tanaka et al. [33] reported a difference of −75.1% in BV/TV, −45.5% in TbTh, +353.6% in TbSp, and −58.1% in TbN between OVX and healthy groups, whereas Irie et al. [34] reported a difference of −14.8% in BV/TV, −13.8% in TbTh, and +21.9% between OVX and healthy groups. In addition to measuring the interradicular septum of the mandibular first molar, Yang et al. [35] also measured the trabecular bone microarchitecture of the region right below the apex of the mesial root of the first molar down to the superior aspect of the incisor socket, and observed significant differences in BV/TV (−17.6%), TbSp (+66.5%), and TbTh (−28.0%) between OVX and healthy groups.

Currently, antiresorptive drugs are front-line drugs in treating osteoporosis; bisphosphonate drugs (e.g., Alendronate, Risedronate, Ibandronate, Zoledronate) are the most common antiresorptive drugs. Previous studies have indicated that this type of drugs is highly effective in treating osteoporosis; however, when used over a long period, they have numerous side effects (e.g., osteonecrosis of the jaw). Osteonecrosis of the jaw can reduce life quality and cause pathologic fracture of the mandible. For treatment of osteoporosis, another approach is anabolic drugs. Previous studies have reported that intermittent PTH enhances bone mass of the lumbar vertebra and long bones. Mashiba et al. [36] sampled part of the tibial midshaft of female New Zealand white rabbits injected with intermittent PTH and used histomorphometry to observe the cortical bone. They showed that intermittent PTH accelerated bone formation and increased the area and strength of the cortical bone. Washimi et al. [31] injected intermittent PTH into ovariectomized rats and used micro-CT to measure the four parameters of the trabecular bone microarchitecture of the distal femoral metaphysis. A group that was administered PTH (10 μg/kg) displayed enhanced BV/TV (48.7%), TbTh (22.7%), TbSp (−24.3%), and TbN (20.5%) following an OVX. The experimental results in this study regarding the femoral head accord with those presented by Washimi et al. [31]. The present study found that the BV/TV and TbTh in the OVX + PTH group increased by 49.8% and 24.0% compared with those in the OVX group, respectively. In addition, the TbSp and TbN changed by −21.0% and 11.1%, which approximates the results reported by Washimi et al. [31]; however, no significant difference was observed in either TbSp or TbN. Similar to previous animal experiments, the present study found that intermittent PTH effectively treated OVX-induced osteoporosis. Clinically, several studies have indicated that intermittent PTH increased BMD in the lumbar vertebra and femur in addition to reducing the risk of bone fractures [11, 37, 38].

In both clinical observations and animal experiments, previous studies have shown that intermittent PTH can prevent osteoporosis of the vertebra body or long bones. However, few studies have investigated the influence of intermittent PTH on the jawbone. Miller et al. [14] began administering PTH (80 μg/kg) once daily to female rats for 10 weeks (5 days per week) one year after they underwent OVX. After 10 weeks of treatment, cross sections of their mandibles were observed through histomorphometry. The results showed that intermittent PTH stimulated bone formation in the mandible. Hunziker et al. [13] also addressed the similar results. In addition, Kawane et al. [39] administered intermittent PTH to ovariectomized rats for 10 weeks and used DXA to measure their mandibular BMD. They observed a significant improvement in BMD in rats with low BMD. Bellido et al. [12] used DXA to observe BMD in the mandibles of ovariectomized rabbits treated with intermittent PTH and reported that the treatment increased their mandibular BMD. Nakajima et al. [40] used confocal laser scanning microscopy and soft X-ray images to observe the bone morphometry of the mandibular condyle of ovariectomized rats treated with various doses of PTH and found that administering PTH (20 μg/kg) three times weekly to an ovariectomized rat attained the most effective enhancement in bone formation.

In the aforementioned studies on using intermittent PTH to treat OVX-induced osteoporosis, histomorphometry, confocal laser scanning microscopy, soft X-ray images, or DXA were used to biochemically examine bone formation or BMD; however, micro-CT was not extensively employed to assess the effect of the treatment on the trabecular bone microarchitecture. The results of the present study show that BV/TV and TbTh were significantly smaller in the OVX group than those in the healthy group. The BV/TV in the OVX + PTH group was larger than that in the OVX group and did not differ significantly from that in the healthy group. Additionally, TbTh increased by 5.18% (the median improved from 0.193 to 0.203 mm); however, the increase was nonsignificant. Notably, compared with the healthy group, the median for the OVVX + PTH group reduced by 15.06%, but the reduction was nonsignificant. Therefore, according to the change in the trabecular bone microarchitecture (i.e., the difference in BV/TV and TbTh), intermittent PTH effectively treated OVC-induced osteoporosis of the mandible.

By comparing the OVX and healthy groups, we found that the OVX group displayed lowered BMD in the trabecular bone compared with that in the healthy group. Regarding the femur and mandible, OVX caused a greater change in BV/TV in the femoral head (−29.9%) than in the jawbone (−25.6%). By contrast, a larger change was observed in TbTh in the jawbone (−19.25%) than in the femoral head (−11.9%). After the rats were treated with intermittent PTH, BV/TV was restored to a healthy state in the femoral head and mandible. The tendencies of TbTh and BV/TV in the femoral head were similar among the three groups. These results accord with the study by Bradbeer et al. [41], who indicated that PTH can increase TbTh. Regarding the mandible, TbTh in the OVX + PTH group (0.203 ± 0.042 mm) was greater than that in the OVX group (0.193 ± 0.007 mm), but the difference was nonsignificant. The reason may be that the region of interest in the femoral head was the trabecular structure, whereas the region of interest in the mandible was the mandibular canal. However, this speculation requires further investigation for confirmation.

The limitations of this study are described as follows. First, rats were used for an animal experiment in the current study. Second, only six rats in each group were used in the experiment. According to the power analysis based on the experimental results, the sample size of each group should be 4, 165, 9 and 9 so that the statistical power would be over 0.8 for post hoc pairwise comparisons using the Mann–Whitney exact tests with the Bonferroni adjustment among the mean differences of BV/TV, TbTh, TbSp, and TbN, respectively. This power analysis will be a reference for the research design of the future studies. Third, this study investigated the trabecular structure only in the lower part of the mandibular first molar and did not explore the effect of intermittent PTH on treating osteoporosis of the maxilla or cortical bone. Fourth, only micro-CT imaging analysis was performed in the present study; neither histologic evaluations nor biomechanical experiments were undertaken. In the future, high-resolution dental CBCT may be used to evaluate the trabecular bone microarchitecture in humans to explore the efficacy of intermittent PTH on treating jawbone deterioration in humans.

## Conclusion

In this study, intermittent PTH was administered to rats to treat OVX-induced osteoporosis. Based on the experimental setup and limitations, the main conclusions of this study regarding the trabecular bone microarchitecture of the mandible and femoral head are offered as follows: (1) The BV/TV and TbTh in the OVX group were lower than those in the healthy group. (2) The BV/TV and TbTh in the OVX + PTH group approximated those in the healthy group, therefore, intermittent PTH displayed high efficacy for treating deteriorated femoral or mandibular bone microarchitecture resulting from loss of ovarian function.

## Abbreviations

BMD: Bone mineral density
BV/TV: Bone volume fraction
dental CBCT: Dental cone-beam CT
DXA: Dual-energy X-ray absorptiometry
IQR: Interquartile range
micro-CT: Microcomputed tomography
OVX: Ovariectomized
PTH: Parathyroid hormone
QCT: Quantitative computed tomography
TbN: Trabecular bone number
TbSp: Trabecular bone separation
TbTh: Trabecular bone thickness
WHO: World Health Organization
